# Ankylostomiasis

**Published:** 1901-10-19

**Authors:** 


### ANKYLOSTOMIASIS.

We have perhaps been too much in the habit of attributing the spread of ankylostomiasis to dirty habits of eating. No doubt this is in fact the principal manner in which the embryos gain access to the body of their victim. "Dirty hands and muddy water and unwashed vegetables are daily factors in the peasant's life, and are probably responsible for much of the infection." Dr. Sandwith,[1] of Cairo, however, has lately called attention to the investigations of Dr. Looss, according to which the embryos of these worms can gain access to the body through the unbroken skin. Dr. Looss' attention was first attracted to this point by the fact that on a drop of water containing a large number of the larvæ of the worm accidentally falling on the cleft between his fingers, this was followed by redness and burning at the spot. On repeating the experiment the same sensations were again produced, and on examining the drop of fluid which was left, countless empty embryo sheaths were found together with a few sluggish embryos. Moreover, Dr. Looss became re-infected with the worms, and he had to undergo prolonged treatment to get rid of them. Some time afterwards an opportunity occurred for bringing the matter to the test of experiment. There was a patient whose leg had to be amputated. An hour before the operation the leg was thoroughly washed with soap, nail brush and water, and then dried; then one drop of water containing many larvæ was dropped on the skin, and was left to itself without being rubbed in. The drop spread out and dried up in ten minutes, producing no redness of the skin. One hour after the drop was applied the limb was amputated. The affected bit of skin was at once removed from the amputated limb and spread out with pins, hardened in alcohol, and then embedded for section cutting. The sections when examined showed that the larvæ had entered the skin principally by the hair follicles. So far as the drop had spread there was hardly a hair follicle free from young ankylostoma. It seems that when once inside the hair follicle they push their way towards the hair papilla, when they pierce the surrounding tissue of the true skin and thus gain access to the system. This discovery, if it should be fully confirmed, greatly adds to the importance of the ankylostoma, and goes far to explain the difficulties which have been met with in checking the spread of its infection.

[1] Journal of Tropical Medicine, Oct. 1.

